# 3-Deazaneplanocin A (DZNep), an Inhibitor of the Histone Methyltransferase EZH2, Induces Apoptosis and Reduces Cell Migration in Chondrosarcoma Cells

**DOI:** 10.1371/journal.pone.0098176

**Published:** 2014-05-22

**Authors:** Nicolas Girard, Céline Bazille, Eva Lhuissier, Hervé Benateau, Antonio Llombart-Bosch, Karim Boumediene, Catherine Bauge

**Affiliations:** 1 Normandie Univ, Caen, France; 2 UNICAEN, EA4652 MILPAT, Caen, France; 3 Service d’Anatomie Pathologique, CHU, Caen, France; 4 Service de Chirurgie Maxillo-faciale, CHU, Caen, France; 5 Pathology Department, University of Valencia, Valencia, Spain; University Hospital of Navarra, Spain

## Abstract

**Objective:**

Growing evidences indicate that the histone methyltransferase EZH2 (enhancer of zeste homolog 2) may be an appropriate therapeutic target in some tumors. Indeed, a high expression of EZH2 is correlated with poor prognosis and metastasis in many cancers. In addition, 3-Deazaneplanocin A (DZNep), an S-adenosyl-L homocysteine hydrolase inhibitor which induces EZH2 protein depletion, leads to cell death in several cancers and tumors. The aim of this study was to determine whether an epigenetic therapy targeting EZH2 with DZNep may be also efficient to treat chondrosarcomas.

**Methods:**

EZH2 expression was determined by immunohistochemistry and western-blot. Chondrosarcoma cell line CH2879 was cultured in the presence of DZNep, and its growth and survival were evaluated by counting adherent cells periodically. Apoptosis was assayed by cell cycle analysis, Apo2.7 expression using flow cytometry, and by PARP cleavage using western-blot. Cell migration was assessed by wound healing assay.

**Results:**

Chondrosarcomas (at least with high grade) highly express EZH2, at contrary to enchondromas or chondrocytes. In vitro, DZNep inhibits EZH2 protein expression, and subsequently reduces the trimethylation of lysine 27 on histone H3 (H3K27me3). Interestingly, DZNep induces cell death of chondrosarcoma cell lines by apoptosis, while it slightly reduces growth of normal chondrocytes. In addition, DZNep reduces cell migration.

**Conclusion:**

These results indicate that an epigenetic therapy that pharmacologically targets EZH2 via DZNep may constitute a novel approach to treat chondrosarcomas.

## Introduction

Polycomb group proteins (PcGs) can remodel chromatin by influencing the degree of compaction, leading to epigenetic gene silencing. In particular, EZH2, the catalytic subunit of Polycomb Repressive Complex 2 (PRC2), induces histone methyltransferase activity primarily by trimethylating histone H3 at lysine 27 (H3K27me3), hence mediating gene silencing. PcGs are crucial in the chromatin control of stem cell self-renewal and differentiation [1]–[7]. They also play a crucial role in malignant progression and are implicated in cancer metastasis [8]. In particular, the methylase EZH2 functions as an oncogene in different human cancers mainly through epigenetic silencing of tumor and metastasis suppressor genes, including E-cadherin [9], RUNX3 [10], SLIT2 [11], DAB2IP [12], FBXO32 [13], and KLF2 [14].

Recent articles showed that EZH2 knockdown results in a significant decrease in cellular proliferation and invasiveness [15]–[18], leading to emerge the concept of epigenetic therapy targeting PcG machinery to cure various tumors, and the development of drugs inhibiting the trimethylation of the lysine 27 on histone 3 (H3K27me3) [19]–[23].

Recently, it has been shown that 3-deazaneplanocin A (DZNep), a carbocyclic analog of adenosine, depletes cellular levels of the PRC2 components, and notably EZH2, and inhibits H3K27me3 [13]. Interestingly, similarly to EZH2 knockdown, DZNep reverts epithelial-to-mesenchymal transition (EMT), and prevents tumor progression, making it a highly promising antimetastatic agent [24]. While the mechanisms and effects of DZNep have been studied in numerous solid tumors and leukemia [13], [25]–[33], less is known about the potential of this compound for sarcomas. In particular its impact on chondrosarcoma, a radio- and chemo-resistant tumor, has never been studied.

Here, we show that high grade chondrosarcomas express EZH2 protein, and that DZNep reduces its expression and subsequently H3K27me3. Interestingly, DZNep treatment induces apoptosis of chondrosarcoma cell lines whereas it has a weak effect on normal chondrocyte, and reduces cell migration, suggesting that targeting EZH2, for instance using DZNep, may be an innovative therapeutic strategy to treat chondrosarcomas.

## Material and Methods

### Reagents

DZNep was provided by R&D Biosystems (Lille, France) and resuspended in phosphate buffered saline (PBS). Inhibitors and propidium iodide were purchased from Sigma and dissolved in PBS. Oligonucleotides were supplied by Eurogentec (Angers, France).

### Human material

This study was approved by the local ethic committee (Comité de protection des personnes Nord Ouest III). Tumoral and normal cartilage was collected from surgical departments of Caen University hospital. All donors signed agreement forms before the surgery, according to local legislations.

### Immunohistochemistry

Multiple specimens of chondrosarcomas (n = 7) or enchondromas (n = 8) were fixed, routinely processed and embedded in paraffin. H&E-stained sections from original block were used to select a representative tumor area. 4-µm sections of non-decalcified chondrosarcomas were prepared from paraffin-embedded tumor blocks and placed on superfrost plus slides. After antigen retrieval with pH 6.0 citrate buffer, immunohistochemistry was performed using an automated immunohistochemical staining processor (Autostainer plus, Dako, Glostrup, Denmark). After incubation with primary antibody EZH2 (Cell signaling, 1∶100), detection was performed using an indirect biotin avidin system, LSABTM2 detection kit (Dako) according to the manufacturer's instructions.

### Cell culture

SW1353 (from ATCC) and CH2879 chondrosarcoma cell line [34] were cultured in Dulbecco's Modified Eagle Medium (DMEM), or Roswell Park Memorial Institute 1640's medium (RPMI 1640) (Lonza AG, Verviers, Belgium), respectively, supplemented with 10% fetal bovine serum (FBS) (Lonza AG), 0.25 µg/ml of fungizone and 10 µM of ciprofloxacin, and then incubated at 37°C in a humidified atmosphere containing 5% CO_2_. Cells were passaged twice a week. Cells were seeded for experiments at 5400 cells/cm^2^ unless indicated otherwise.

The normal cartilage was obtained from biopsy of nasal cartilage. Chondrocytes were released by digestion with XIV Pronase (2 mg/ml for 30 minutes, Sigma-Aldrich, St Quentin Fallavier, France) and type I collagenase (2 mg/ml for 15 hours, Invitrogen, Cergy-Pontoise, France). The cells were incubated in Dulbecco's modified Eagle's medium (DMEM), supplemented with 10% FBS, 0.25 µg/ml fungizone and 10 µM of ciprofloxacin, and then incubated at 37°C in a humidified atmosphere containing 5% CO_2_.

### Protein extraction and Western blot

Cells were rinsed with phosphate buffered saline (PBS) to remove residual FBS and scraped into RIPA lysis buffer (Tris-HCl 50 mM pH 7,5; IGEPAL 1%; NaCl 150 mM; EGTA 1 mM; NaF 1 mM) supplemented with phosphatase (NA_3_VO_4_ 10 µL/ml) and protease inhibitors (leupeptin 1 µl/ml, aprotinin 1 µl/ml, pepstatin 1 µl/ml and phenylmethylsulfonyl fluoride 4 µl/ml). Proteins (20 to 50 µg) were resolved by SDS-PAGE and transferred to polyvinylidene difluoride membranes (Bio-Rad). The membranes were incubated with 10% nonfat milk or 1% bovin serum albumin (BSA) 1 hour at room temperature, incubated with primary antibodies overnight at 4°C at the appropriated dilutions (EZH2, 1∶1000 dilution, Cell signaling, catalog no. 5246; PARP, 1∶1000 dilution, Cell signaling, catalog no. 9542; H3K27me3, 1∶1000 dilution, Abcam, catalog no. ab6002; actin, 1∶200 dilution, Santa cruz biotechnology, catalog no. sc-8432; H3, 1∶2000 dilution, Abcam, catalog no. ab1791). The membranes were washed with TBS-T and probed with a corresponding secondary antibody conjugated to horsoradish peroxydase in TBS-T at room temperature (goat anti-mouse IgG-HRP and goat anti-rabbit IgG-HRP, 1∶10000, Santa cruz biotechnology). Signals were revealed with Western Lightning ® Plus-ECL (Perkin Elmer) and exposed to X-ray film (Kodak). Actin or histone H3 were used to verify that similar amounts of protein were loaded in all lanes.

### RNA isolation and real-time reverse transcription-polymerase chain reaction (RT-PCR)

Total RNA was extracted with Trizol reagent according to the manufacturer's condition (Invitrogen). Samples (2 µg) were treated with DNAse I (Invitrogen, Cergy-Pontoise, France) and the reverse transcriptase was effected with oligo dT and Moloney murine leukemia virus reverse transcriptase. Complementary DNA was diluted (1∶100) and stocked at −20°Cpending for PCR. This product (5 µL) was mixed with appropriated reverse and forward primers and SYBR Green PCR Master Mix (Applied Biosystems, Villebon sur Yvette, France) in 15 µL volume final. RT-PCR was run in an ABI Prism 7000 sequence detection system apparatus. Relative expression was calculated according to the 2^−ΔΔCt^ method [35].

### Cell growth experiment

Cells were seeded at 750 cells/cm^2^ and treated with DZNep (1 µM) for 14 days. The medium was changed twice during the treatment. Adherent cells were counted each indicated time (4, 7, 9, 14 days).

### Flow cytometry

Cells were treated with DZNep (1 µM) for 7 days. Then, cells were washed with PBS, treated with trypsin-EDTA (Lonza AG, Verviers, Belgium) and fixed with 70% ethanol at −20°C and conserved at 4°C. For analysis, cells were washed twice with PBS and resuspended in 20 µg/ml RNase (Invitrogen, Cergy-Pontoise, France) and 50 µg/ml propidium iodide (IP) (Sigma Aldrich, St Quentin Fallavier, France) to label the DNA. DNA content was measured using Gallios (Beckman Coulter, Villepinte, France) on the technical platform of SFR 146 (Structure Federative de Recherche 146, Caen, France). Results were analyzed with Kaluza software.

To study apoptosis, living cells were stained with Apo 2.7-PE antibody according to the manufacturer's condition (Beckman Coulter, Villepinte, France). The expression of Apo 2.7 was detected using Gallios on the technical platform of the SFR 146.

### Wound healing assay

Cells were seeded at 40000 cells/cm^2^, treated with DZNep (1 µM) for 5 days. At day 4, a straight scratch was made with a 200 µl pipette tip and the wound was photographed under the microscope. After 24 h, cells were stained with crystal violet 0.1% for 10 minutes and photographed under the microscope. The area of the remaining scratch was calculated using Image J software (http://rsb.info.nih.gov/ij/).

### Statistical analysis

For in vitro experiments, three different experiments were performed. The values are means ± SEM. Statistical significancy was calculated with Student's t test.

## Results

### EZH2 is expressed in chondrosarcomas

**Figure 1:**
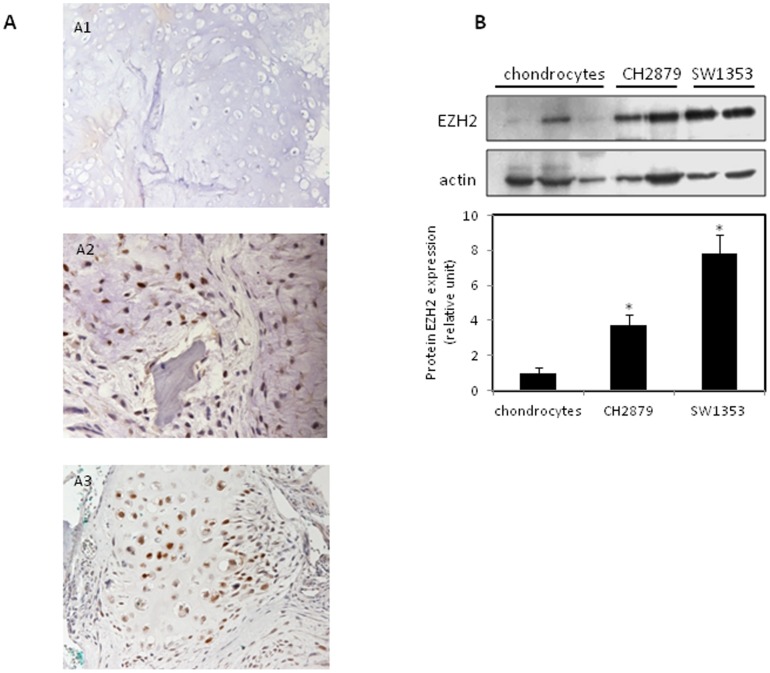
EZH2 is expressed in chondrosarcoma. A. EZH2 expression was analyzed by immunostaining in enchondromas (n = 8) (A1), or grade 2 and 3 chondrosarcomas (n = 7; 6 samples were positive for EZH2 staining) (A2 and A3 respectively). B. EZH2 expression was analyzed in two batches of both SW1353 and CH2879 chondrosarcoma cell lines and normal chondrocytes by Western blot. Actin was used to compare protein loading.

First, we investigated whether EZH2 is expressed in chondrosarcomas. Immunohistochemistry analysis from patient biopsies showed that EZH2 is expressed in nucleus of high grade chondrosarcomas (for 6/7 samples). Interestingly, we could not detect EZH2 in all enchondromas tested (n = 8) (figure 1A). Furthermore, by Western-Blot, we found that EZH2 expression was higher in chondrosarcoma cells than normal chondrocytes (figure 1B). Since EZH2 is highly expressed in chondrosarcomas, we hypothesized that these tumors may be a sensitive to EZH2 inhibitors, such as DZNep.

### DZNep inhibits EZH2 and reduces H3K27me3

**Figure 2:**
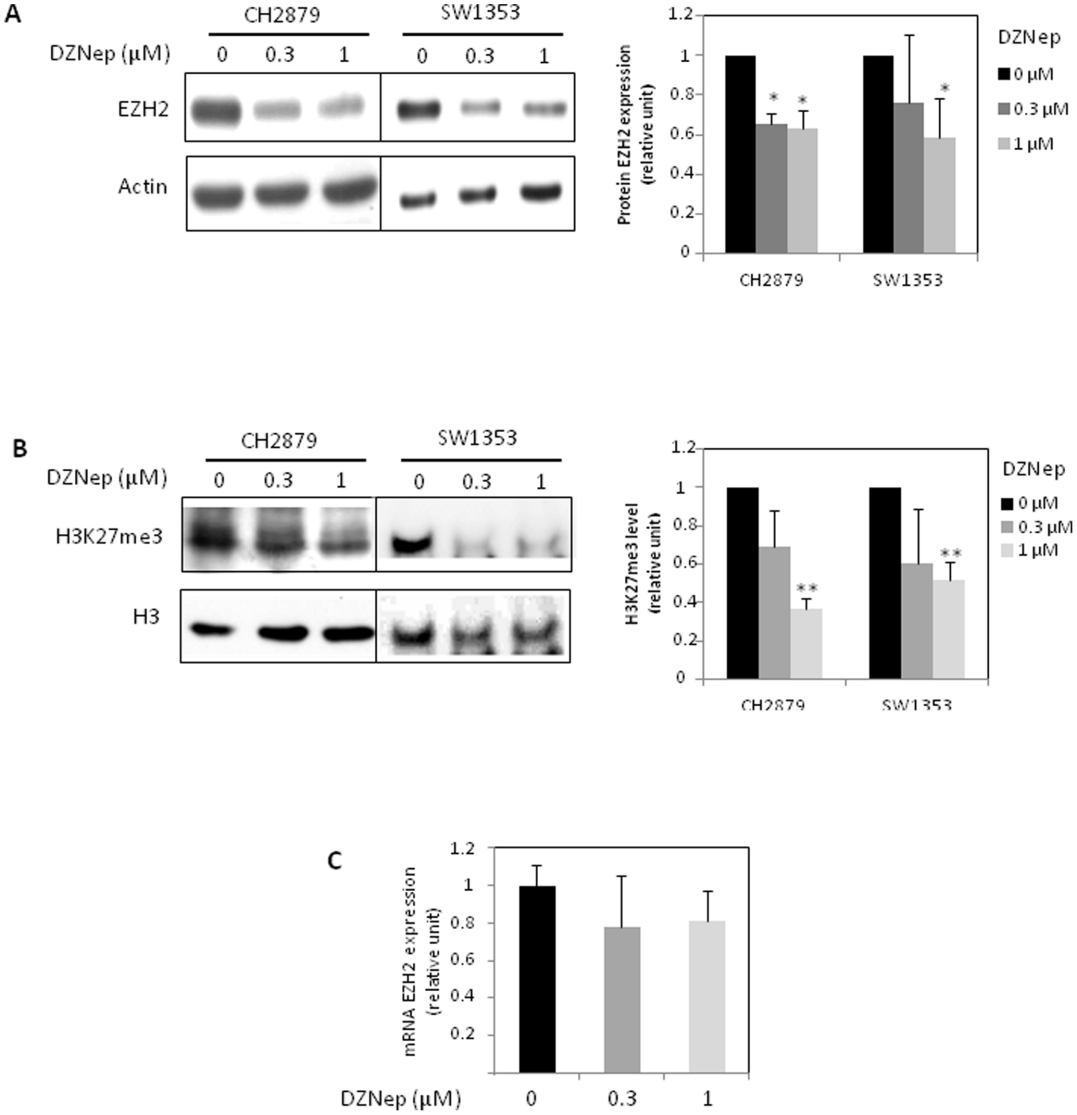
DZNep reduces EZH2 protein expression and H3K27me3 and but not mRNA. SW1353 and CH2879 cells were treated with DZNep (1 µM) for 72 h. A) EZH2 protein expression was analyzed by Western blot. Histograms represent the quantification of three independent experiments after normalization with actin. B) H3K27me3 was also analyzed by Western blot. H3 was used to compare protein loading. Histograms represent the quantification of three independent experiments after normalization with H3. C) EZH2 mRNA expression was analyzed by RT-PCR from CH2879 treated with DZNep for 72 h. Data were expressed as means ± SEM.

As expected, DZNep treatment reduced EZH2 protein level, and subsequently H3K27me3 level in two chondrosarcoma cell lines, CH2879 and SW1353 (figure 2A and B). To investigate whether decreased levels of EZH2 protein resulted from transcriptional regulation, we performed quantitative real time-PCR analysis. DZNep treatment had no effect on EZH2 expression at mRNA level (figure 2C).

### DZNep selectively induces cytotoxicity in cancerous but not normal cartilage cells

**Figure 3:**
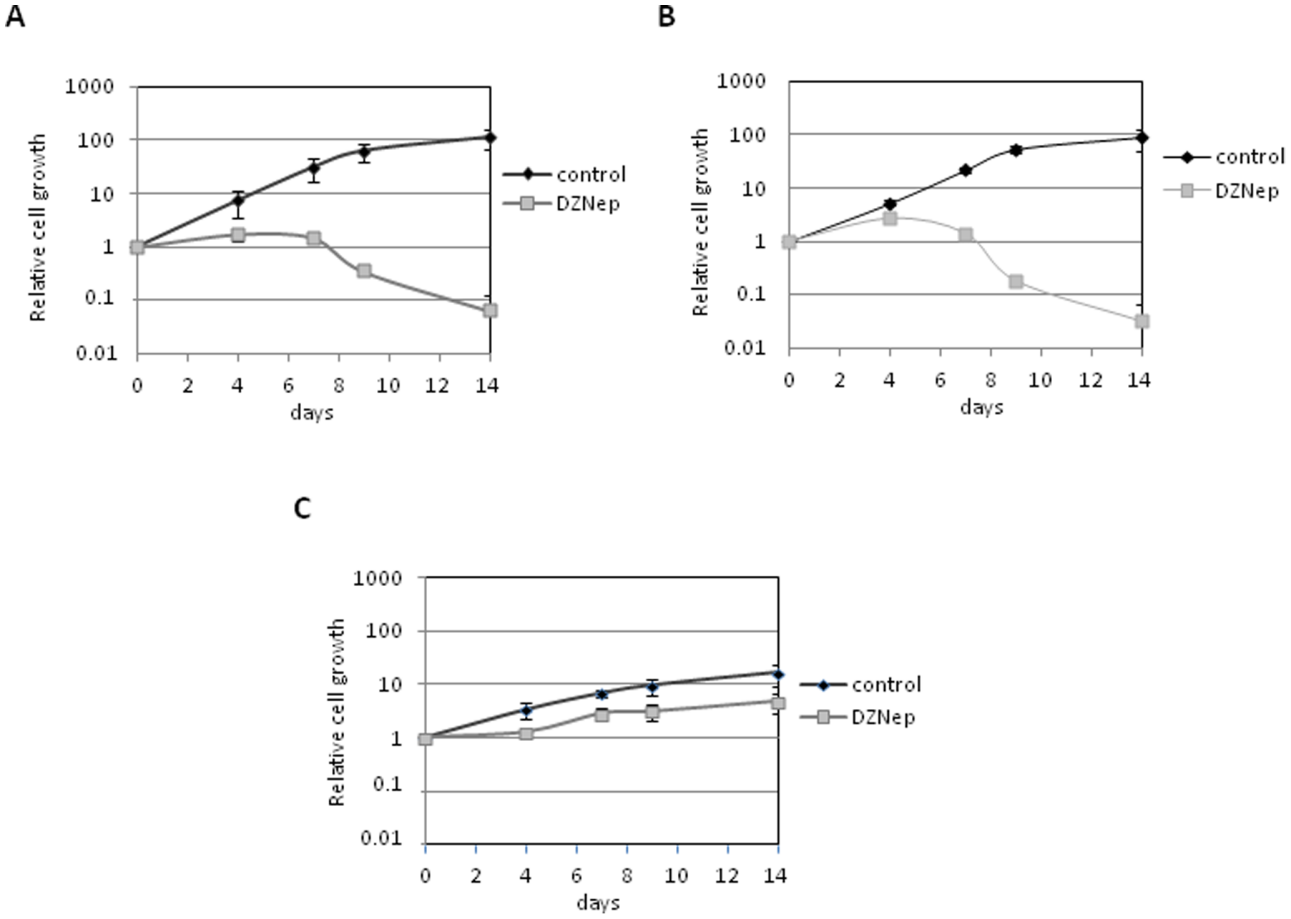
DZNep induces death in chondrosarcoma but not chondrocytes. CH2878 cells (A), SW1353 (B), or chondrocytes (C) were treated with DZNep (1 µM) for 14 days. Treatment was renewed at each medium changes (at days 4, 7 and 10), and adherent cells regularly counted. The results of three independent experiments are shown. Data are expressed as means ± SEM.

Furthermore, we evaluated the effect of DZNep treatment on growth of chondrosarcoma cells and chondrocytes. DZNep induced death of chondrosarcoma cells with a delay (figure 3A), whereas it slightly decreased chondrocyte growth (figure 3B). These results confirm the potential therapeutic of DZNep on chondrosarcoma.

### DZNep induces apoptosis in chondrosarcomas

**Figure 4:**
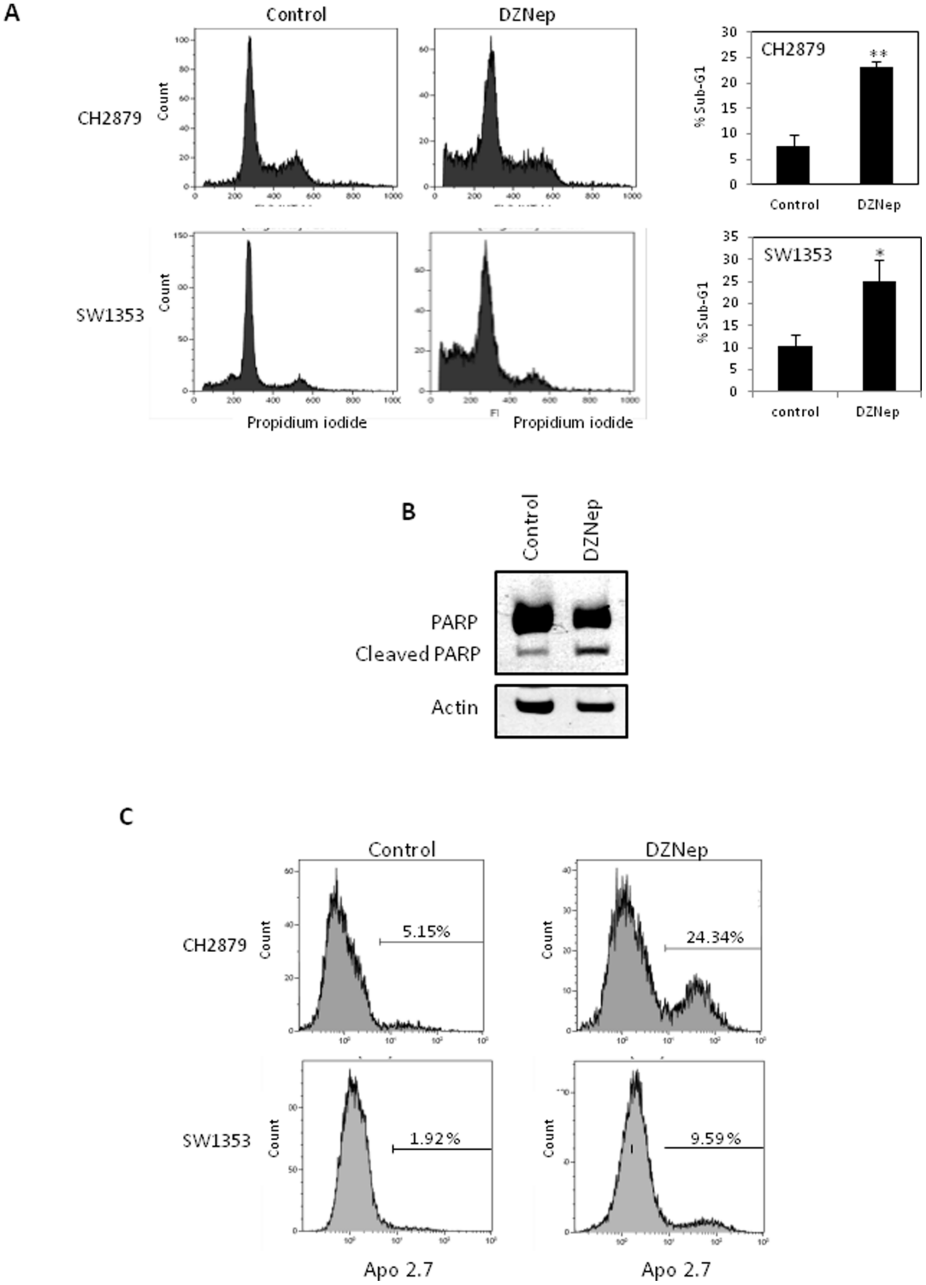
DZNep induces apoptosis in SW1353 and CH2879 chondrosarcoma cell lines. Cells were treated with DZNep (1 µM) for 7 days (A and C) or 5 days (B). A) At day 7, cells were fixed and cell cycle determined by flow cytometry. Histograms represent the sub-G1 phase percentage from three independent experiments. B) At day 5, proteins were extracted and PARP protein expression was analyzed by Western blot. C) At day 7, cells were stained with Apo 2.7 antibody coupled to phycoerythrin and analyzed by flow cytometry.

We then examined whether DZNep affects cell cycle. DZNep treatment increased sub-G1 peak, without visible arrest in cell cycle (figure 4A). DZNep also induced PARP cleavage and Apo 2.7 protein expression (figures 4 B and C) demonstrating an apoptotic death of chondrosarcomas.

### DZNep reduces cell migration

**Figure 5:**
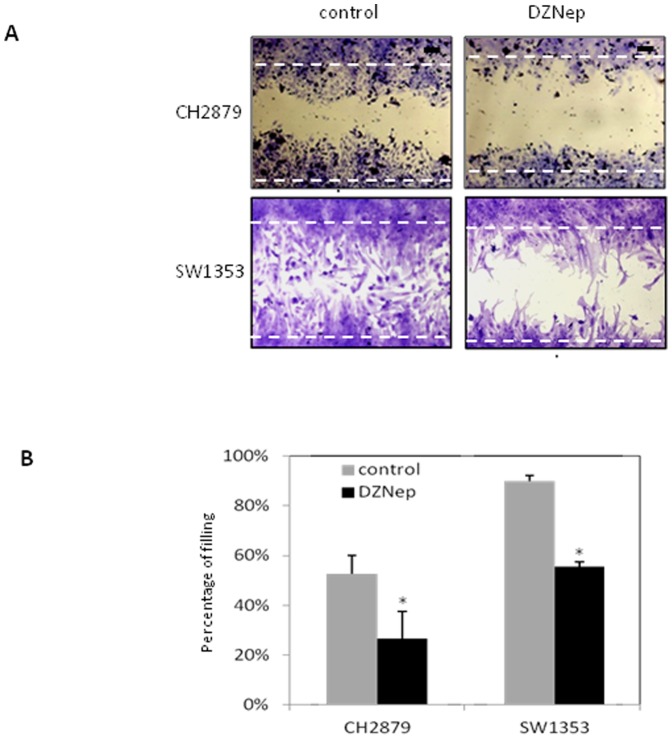
DZNep reduces SW1353 and CH2879 chondrosarcoma migration. Cells were pretreated 4 days with DZNep (1 µM) and a straight scratch was made in individual 6-wells dishes with a 200 µL pipette tip. A) Microscopic observations were recorded 24 hours after scratching the cell surface. Dotted lines showed the initial mark of the scratch. B) Graph represents the percentage of filling 24 hours after the wound. Data were expressed as means ± SEM.

Finally, the effect of DZNep on migration was also examined by wound healing assay (figure 5). We found that compared to control, DZNep reduced the migration of chondrosarcomas.

## Discussion

With the advent of systemic chemotherapy in the management of mesenchymal malignancies such as osteosarcoma and Ewing's sarcoma, there has been an increase in the long-term survival of patients. In contrast, chondrosarcomas continue to have a poor prognosis owing to the absence of an effective therapy [36]–[38]. Identifying new drugs that enables to reduce chondrosarcoma growth may improve survival of patients. Here, we identified, 3-Deazaneplanocin A (DZNep), a small molecule EZH2 inhibitor [13], [39], as a putative treatment of chondrosarcomas. Indeed, we show that DZNep treatment significantly reduces the EZH2 protein and H3K27 trimethylation level, and induces chondrosarcoma death by apoptosis while it decreases their migration ability.

First, we showed for the first time, that human chondrosarcomas, but not enchondromas, express EZH2 protein. In addition, EZH2 level was more elevated in a grade II and III chondrosarcoma cell lines, SW1353 and CH2879, than in chondrocytes. This agrees with observations in other tumors showing that EZH2 is overexpressed in cancers, including melanoma, lymphoma, and breast and prostate cancers [13], [27], [33], [40]. This high expression of EZH2 is related to poor prognostic in these cancers and tumors [8], [41], [42]. The potential use of EZH2 expression for improving diagnostic of chondrosarcomas and the correlation between its expression and tumor grade or prognostic for patients is still in process.

Furthermore, we show for the first time that DZNep is efficient to reduce chondrosarcoma growth, survival and migration, *in vitro*. Numerous studies suggest that DZNep induces death in tumoral cells though EZH2 downregulation and H3K27me3 reduction [13], [30], [31], [43]. In our chondrosarcoma model, we also found that DZNep reduces EZH2 at protein level (but not at mRNA level) and subsequently decreases H3K27me3. This discrepancy between mRNA and protein levels has ever been observed with other tumoral cells [13], [44], and can be explained by the mechanism by which DZNep acts on EZH2. Indeed, DZNep acts indirectly by inhibiting an S-adenosylhomocysteine (SAH) hydrolase, which induce an accumulation of SAH, leading to the degradation of EZH2 protein [39].

However, at this point, we cannot ascertain that chondrosarcoma death induced by DZNep is directly due to EZH2 inhibition. Indeed, DZNep is an AdoHcy hydrolase inhibitor and is able to inhibit methylation of another repressive histone marks, such as H4-K20 methylation [13]. More recently, it has been reported, in MCF7 cells, that DZNep also causes a global decrease in most histone modifications, except for H3K9me3 and H3K37me3, implicating that DZNep is effective in decreasing histone modifications with both repressive and active chromatin markers, in a non-selective manner [39], [45]. Therefore, the molecular mechanisms of DZNep might be more complex than our current knowledge base, which associates the downregulation of EZH2 with the ability of DZNep to induce tumor cell death.

Contrary to the majority of tumoral cells, we found that DZNep exerts cytotoxicity in chondrosarcomas with a delay. This was also observed in pancreatic tumors [46]. However, similarly to a number of tumor cells, we found that DZNep induced apoptosis in chondrosarcomas, suggesting that the mechanism of death is shared with all tumors cells, whereas here the effect was delayed. We hypothesize that this delay may be due to a slower proliferation kinetic of chondrosarcomas compared to other tumoral cells. Interestingly, normal chondrocytes only show a slight decrease of their growth upon DZNep treatment. Similarly, other report also show that DZNep does not induce apoptosis in normal cells, making it a promising drug candidate for anti-cancer treatment, in particular to treat radioresistant and chemoresistant tumors such as chondrosarcomas.

In conclusion, in this study, we describe the effect of an AdoHcy hydrolase inhibitor, DZNep, on EZH2 expression and subsequent H3K27me3 in chondrosarcomas, as well as its ability to preferentially induce death by apoptosis in tumoral cartilage cells than normal chondrocytes.

## References

[1] Aloia L, Stefano BD, Croce LD (2013) Polycomb complexes in stem cells and embryonic. Development 140: 2525–2534 10.1242/dev.091553 23715546

[2] Pereira CF, Piccolo FM, Tsubouchi T, Sauer S, Ryan NK, et al (2010) ESCs require PRC2 to direct the successful reprogramming of differentiated cells toward pluripotency. Cell Stem Cell 6: 547–556 10.1016/j.stem.2010.04.013 20569692

[3] Ding X, Wang X, Sontag S, Qin J, Wanek P, et al.. (2013) The Polycomb Protein Ezh2 Impacts on iPS Cell Generation. Stem Cells Dev: 131210220315000. doi:10.1089/scd.2013.0267 10.1089/scd.2013.0267 PMC3996971 24325319

[4] Fragola G, Germain P-L, Laise P, Cuomo A, Blasimme A, et al (2013) Cell reprogramming requires silencing of a core subset of polycomb targets. PLoS Genet 9: e1003292 10.1371/journal.pgen.1003292 23468641 PMC3585017

[5] Chen Y-H, Hung M-C, Li L-Y (2012) EZH2: a pivotal regulator in controlling cell differentiation. Am J Transl Res 4: 364–375. 23145205 PMC3493026

[6] Juan AH, Derfoul A, Feng X, Ryall JG, Dell'Orso S, et al (2011) Polycomb EZH2 controls self-renewal and safeguards the transcriptional identity of skeletal muscle stem cells. Genes Dev 25: 789–794 10.1101/gad.2027911 21498568 PMC3078704

[7] Wang L, Jin Q, Lee J-E, Su I, Ge K (2010) Histone H3K27 methyltransferase Ezh2 represses Wnt genes to facilitate adipogenesis. Proc Natl Acad Sci U S A 107: 7317–7322 10.1073/pnas.1000031107 20368440 PMC2867706

[8] Varambally S, Dhanasekaran SM, Zhou M, Barrette TR, Kumar-Sinha C, et al (2002) The polycomb group protein EZH2 is involved in progression of prostate cancer. Nature 419: 624–629 10.1038/nature01075 12374981

[9] Cao Q, Yu J, Dhanasekaran SM, Kim JH, Mani R-S, et al (2008) Repression of E-cadherin by the Polycomb Group Protein EZH2 in Cancer. Oncogene 27: 7274–7284 10.1038/onc.2008.333 18806826 PMC2690514

[10] Fujii S, Ito K, Ito Y, Ochiai A (2008) Enhancer of Zeste Homologue 2 (EZH2) Down-regulates RUNX3 by Increasing Histone H3 Methylation. J Biol Chem 283: 17324–17332 10.1074/jbc.M800224200 18430739 PMC2427338

[11] Yu J, Cao Q, Yu J, Wu L, Dallol A, et al (2010) The neuronal repellent SLIT2 is a target for repression by EZH2 in prostate cancer. Oncogene 29: 5370–5380 10.1038/onc.2010.269 20622896 PMC2948081

[12] Min J, Zaslavsky A, Fedele G, McLaughlin SK, Reczek EE, et al (2010) An oncogene-tumor suppressor cascade drives metastatic prostate cancer by coordinately activating Ras and NF-? B. Nat Med 16: 286–294 10.1038/nm.2100 20154697 PMC2903662

[13] Tan J, Yang X, Zhuang L, Jiang X, Chen W, et al (2007) Pharmacologic disruption of Polycomb-repressive complex 2-mediated gene repression selectively induces apoptosis in cancer cells. Genes Dev 21: 1050–1063 10.1101/gad.1524107 17437993 PMC1855231

[14] Taniguchi H, Jacinto FV, Villanueva A, Fernandez AF, Yamamoto H, et al (2012) Silencing of Kruppel-like factor 2 by the histone methyltransferase EZH2 in human cancer. Oncogene 31: 1988–1994 10.1038/onc.2011.387 21892211 PMC3325596

[15] Chen Y, Xie D, Yin Li W, Man Cheung C, Yao H, et al (2010) RNAi targeting EZH2 inhibits tumor growth and liver metastasis of pancreatic cancer in vivo. Cancer Lett 297: 109–116 10.1016/j.canlet.2010.05.003 20684863

[16] Eskander RN, Ji T, Huynh B, Wardeh R, Randall LM, et al (2013) Inhibition of enhancer of zeste homolog 2 (EZH2) expression is associated with decreased tumor cell proliferation, migration, and invasion in endometrial cancer cell lines. Int J Gynecol Cancer Off J Int Gynecol Cancer Soc 23: 997–1005 10.1097/IGC.0b013e318296a265 PMC3694282 23792601

[17] Li H, Cai Q, Godwin AK, Zhang R (2010) Enhancer of zeste homolog 2 promotes the proliferation and invasion of epithelial ovarian cancer cells. Mol Cancer Res MCR 8: 1610–1618 10.1158/1541-7786.MCR-10-0398 21115743 PMC3059727

[18] Ougolkov AV, Bilim VN, Billadeau DD (2008) Regulation of Pancreatic Tumor Cell Proliferation and Chemoresistance by the Histone Methyltransferase EZH2. Clin Cancer Res Off J Am Assoc Cancer Res 14: 6790–6796 10.1158/1078-0432.CCR-08-1013 PMC2690708 18980972

[19] Kelly TK, De Carvalho DD, Jones PA (2010) Epigenetic Modifications as Therapeutic Targets. Nat Biotechnol 28. Available: http://www.ncbi.nlm.nih.gov/pmc/articles/PMC3022972/. Accessed 2013 December 19. 10.1038/nbt.1678 PMC3022972 20944599

[20] McCabe MT, Ott HM, Ganji G, Korenchuk S, Thompson C, et al (2012) EZH2 inhibition as a therapeutic strategy for lymphoma with EZH2-activating mutations. Nature 492: 108–112 10.1038/nature11606 23051747

[21] Qi W, Chan H, Teng L, Li L, Chuai S, et al (2012) Selective inhibition of Ezh2 by a small molecule inhibitor blocks tumor cells proliferation. Proc Natl Acad Sci U S A 109: 21360–21365 10.1073/pnas.1210371110 23236167 PMC3535655

[22] Wagner T, Jung M (2012) New lysine methyltransferase drug targets in cancer. Nat Biotechnol 30: 622–623 10.1038/nbt.2300 22781684

[23] Zhao X, Lwin T, Zhang X, Huang A, Wang J, et al (2013) Disruption of the MYC-miRNA-EZH2 loop to suppress aggressive B-cell lymphoma survival and clonogenicity. Leukemia 27: 2341–2350 10.1038/leu.2013.94 23538750 PMC4015113

[24] Crea F, Paolicchi E, Marquez VE, Danesi R (2012) Polycomb genes and cancer: time for clinical application? Crit Rev Oncol Hematol 83: 184–193 10.1016/j.critrevonc.2011.10.007 22112692

[25] Chase A, Cross NCP (2011) Aberrations of EZH2 in Cancer. Clin Cancer Res 17: 2613–2618 10.1158/1078-0432.CCR-10-2156 21367748

[26] Cheng LL, Itahana Y, Lei ZD, Chia N-Y, Wu Y, et al (2012) TP53 genomic status regulates sensitivity of gastric cancer cells to the histone methylation inhibitor 3-deazaneplanocin A (DZNep). Clin Cancer Res Off J Am Assoc Cancer Res 18: 4201–4212 10.1158/1078-0432.CCR-12-0036 22675170

[27] Choudhury SR, Balasubramanian S, Chew YC, Han B, Marquez VE, et al (2011) (-)-Epigallocatechin-3-gallate and DZNep reduce polycomb protein level via a proteasome-dependent mechanism in skin cancer cells. Carcinogenesis 32: 1525–1532 10.1093/carcin/bgr171 21798853 PMC3179425

[28] Crea F, Hurt EM, Mathews LA, Cabarcas SM, Sun L, et al (2011) Pharmacologic disruption of Polycomb Repressive Complex 2 inhibits tumorigenicity and tumor progression in prostate cancer. Mol Cancer 10: 40 10.1186/1476-4598-10-40 21501485 PMC3100246

[29] Crea F, Fornaro L, Bocci G, Sun L, Farrar WL, et al (2012) EZH2 inhibition: targeting the crossroad of tumor invasion and angiogenesis. Cancer Metastasis Rev 31: 753–761 10.1007/s10555-012-9387-3 22711031

[30] Hayden A, Johnson PWM, Packham G, Crabb SJ (2011) S-adenosylhomocysteine hydrolase inhibition by 3-deazaneplanocin A analogues induces anti-cancer effects in breast cancer cell lines and synergy with both histone deacetylase and HER2 inhibition. Breast Cancer Res Treat 127: 109–119 10.1007/s10549-010-0982-0 20556507

[31] Puppe J, Drost R, Liu X, Joosse SA, Evers B, et al (2009) BRCA1-deficient mammary tumor cells are dependent on EZH2 expression and sensitive to Polycomb Repressive Complex 2-inhibitor 3-deazaneplanocin A. Breast Cancer Res BCR 11: R63 10.1186/bcr2354 19709408 PMC2750125

[32] Suvà M-L, Riggi N, Janiszewska M, Radovanovic I, Provero P, et al (2009) EZH2 Is Essential for Glioblastoma Cancer Stem Cell Maintenance. Cancer Res 69: 9211–9218 10.1158/0008-5472.CAN-09-1622 19934320

[33] Zhou J, Bi C, Cheong L-L, Mahara S, Liu S-C, et al (2011) The histone methyltransferase inhibitor, DZNep, up-regulates TXNIP, increases ROS production, and targets leukemia cells in AML. Blood 118: 2830–2839 10.1182/blood-2010-07-294827 21734239

[34] Gil-Benso R, Lopez-Gines C, Lopez-Guerrero JA, Carda C, Callaghan RC, et al. (n.d.) Establishment and Characterization of a Continuous Human Chondrosarcoma Cell Line, ch-2879: Comparative Histologic and Genetic Studies with Its Tumor of Origin. Lab Invest 83: 877–887. 10.1097/01.lab.0000073131.34648.ea 12808123

[35] Livak KJ, Schmittgen TD (2001) Analysis of relative gene expression data using real-time quantitative PCR and the 2(-Delta Delta C(T)) Method. Methods San Diego Calif 25: 402–408 10.1006/meth.2001.1262 11846609

[36] Duchman KR, Lynch CF, Buckwalter JA, Miller BJ (2014) Estimated Cause-specific Survival Continues to Improve Over Time in Patients With Chondrosarcoma. Clin Orthop. doi:10.1007/s11999-014-3600-3 10.1007/s11999-014-3600-3 PMC4079873 24706044

[37] Italiano A, Mir O, Cioffi A, Palmerini E, Piperno-Neumann S, et al (2013) Advanced chondrosarcomas: role of chemotherapy and survival. Ann Oncol Off J Eur Soc Med Oncol ESMO 24: 2916–2922 10.1093/annonc/mdt374 PMC3811906 24099780

[38] Angelini A, Guerra G, Mavrogenis AF, Pala E, Picci P, et al (2012) Clinical outcome of central conventional chondrosarcoma. J Surg Oncol 106: 929–937 10.1002/jso.23173 22649023

[39] Miranda TB, Cortez CC, Yoo CB, Liang G, Abe M, et al (2009) DZNep Is a Global Histone Methylation Inhibitor that Reactivates Developmental Genes Not Silenced by DNA Methylation. Mol Cancer Ther 8: 1579–1588 10.1158/1535-7163.MCT-09-0013 19509260 PMC3186068

[40] Holm K, Grabau D, Lövgren K, Aradottir S, Gruvberger-Saal S, et al (2012) Global H3K27 trimethylation and EZH2 abundance in breast tumor subtypes. Mol Oncol 6: 494–506 10.1016/j.molonc.2012.06.002 22766277 PMC5528390

[41] Bachmann IM, Halvorsen OJ, Collett K, Stefansson IM, Straume O, et al (2006) EZH2 expression is associated with high proliferation rate and aggressive tumor subgroups in cutaneous melanoma and cancers of the endometrium, prostate, and breast. J Clin Oncol Off J Am Soc Clin Oncol 24: 268–273 10.1200/JCO.2005.01.5180 16330673

[42] Mallen-St. Clair J, Soydaner-Azeloglu R, Lee KE, Taylor L, Livanos A, et al (2012) EZH2 couples pancreatic regeneration to neoplastic progression. Genes Dev 26: 439–444 10.1101/gad.181800.111 22391448 PMC3305982

[43] Chiba T, Suzuki E, Negishi M, Saraya A, Miyagi S, et al (2012) 3-Deazaneplanocin A is a promising therapeutic agent for the eradication of tumor-initiating hepatocellular carcinoma cells. Int J Cancer 130: 2557–2567 10.1002/ijc.26264 21717453

[44] Fujiwara T, Saitoh H, Inoue A, Kobayashi M, Okitsu Y, et al (2014) 3-Deazaneplanocin A (DZNep), an Inhibitor of S-Adenosylmethionine-dependent Methyltransferase, Promotes Erythroid Differentiation. J Biol Chem 289: 8121–8134 10.1074/jbc.M114.548651 24492606 PMC3961643

[45] Varier RA, Timmers HTM (2011) Histone lysine methylation and demethylation pathways in cancer. Biochim Biophys Acta BBA - Rev Cancer 1815: 75–89 10.1016/j.bbcan.2010.10.002 20951770

[46] Hung SW, Mody H, Marrache S, Bhutia YD, Davis F, et al. (2013) Pharmacological Reversal of Histone Methylation Presensitizes Pancreatic Cancer Cells to Nucleoside Drugs: In Vitro Optimization and Novel Nanoparticle Delivery Studies. PLoS ONE 8. Available: http://www.ncbi.nlm.nih.gov/pmc/articles/PMC3735519/. Accessed 2013 September 10. 10.1371/journal.pone.0071196 PMC3735519 23940717

